# BCAA metabolism in cancer progression and therapy resistance: The balance between fuel and cell signaling

**DOI:** 10.3389/fphar.2025.1595176

**Published:** 2025-05-14

**Authors:** Yi Zhou, Jiahui Kou, Wenjin Li, Yuyao Wang, Xingxing Su, Hongguang Zhang

**Affiliations:** ^1^ Departments of Thoracic Surgery, First Hospital of Shanxi Medical University, Taiyuan, China; ^2^ School of Basic Medicine, Shanxi Medical University, Taiyuan, China; ^3^ Shunyi Maternal and Children’s Hospital of Beijing Children’s Hospital, Beijing, China

**Keywords:** branched-chain amino acids (BCAAs), tumor progression, metabolic reprogramming, therapy resistance, the tumor microenvironment (TME)

## Abstract

Branched-chain amino acids (BCAAs), including leucine, isoleucine, and valine, play a crucial role in cellular metabolism and signaling. Recent studies have demonstrated that BCAA metabolic reprogramming is a key driver of tumor progression and treatment resistance in various cancers. BCAA metabolism supports cancer cell growth, survival, and proliferation by modulating pathways such as mTOR signaling and oxidative stress responses. By promoting immunosuppressive conditions and increasing the survival rate of cancer stem cells (CSCs), BCAAs contribute to immune evasion and resistance to therapies such as chemotherapy and immune checkpoint inhibitors. This article explores the different metabolic reprogramming patterns of BCAAs in various tumors and introduces BCAA-related metabolic targets for overcoming tumor resistance, offering new directions for precision cancer treatment, reducing resistance, and improving patient outcomes.

## 1 Introduction

Cell metabolism is a fundamental characteristic for maintaining life activities. The growth, differentiation, death, and stress responses of cells require regulation centered around metabolites and metabolic enzymes (Pavlova et al., 2022; Martínez-Reyes and Chandel, 2021; Stine et al., 2022). Recent studies have shown that metabolites can act as signaling molecules to regulate cellular signal transduction and participate in various biological processes such as intercellular communication and epigenetic regulation (Bergers and Fendt, 2021; Wu et al., 2023; Zanotelli et al., 2021). Compared to normal physiological activities, tumor metabolism is a highly complex process that involves an imbalance of multiple metabolites and the reshaping of metabolic pathways during tumor development. Tumor cell metabolism requires the support of various nutrients, including glucose, amino acids, and fatty acids. For example, under normal oxygen conditions, tumor cells, unlike normal cells, still rely heavily on glycolysis to consume large amounts of glucose and produce lactic acid—a phenomenon known as the “Warburg effect” (Zhong et al., 2022). Tumors are widely recognized as metabolic diseases, and metabolic reprogramming is one of the key characteristics of tumors (Xia et al., 2021). Metabolic reprogramming enables tumor cells to adjust their metabolic patterns in response to various stimuli and stressors in the microenvironment, thereby enhancing their survival and proliferation (Martínez-Reyes and Chandel, 2021). The occurrence of tumor metabolic reprogramming may result from the activation or mutation of oncogenes and tumor suppressor genes, which alters the expression and activity of key metabolic enzymes in metabolic signaling pathways, leading to metabolic reprogramming and tumor progression (Xu D. et al., 2021).

Branched-chain amino acids (BCAAs) are essential amino acids for human nutrition and include three amino acids with branched side chains: leucine, isoleucine, and valine. These three amino acids not only form the basic building blocks of proteins but also play critical roles in cellular signaling pathways, energy metabolism, and immune regulation, influencing tumor development and progression (Sivanand and Vander Heiden, 2020; Qian et al., 2023; Ma et al., 2024).

The tumor microenvironment (TME) is a complex cellular environment in which tumor cells reside, composed of various cell types and extracellular components surrounding the tumor cells (de Visser and Joyce, 2023). Cells and extracellular components in the TME interact with tumor cells, promoting their proliferation and invasion while reducing drug permeability. Immune cells are a key component of the TME, and amino acids are essential for protein synthesis and play a role in various physiological activities and immune system regulation (Kao et al., 2022; Leone and Powell, 2020). The reshaping of amino acid metabolism provides energy and raw materials for tumor growth and acts as signaling molecules regulating tumor development (Muthusamy et al., 2020). They also function in maintaining cellular redox balance, driving nucleotide synthesis, and generating energy (Raffel et al., 2017). Particularly, in different types of tumors, selectively inhibiting tumor progression can be achieved by limiting specific amino acid metabolism (Ericksen et al., 2019). Tumor resistance remains a major challenge in cancer treatment, leading to treatment failure and disease progression (O’Donnell et al., 2019). Metabolic reprogramming-induced tumor resistance mediated by the tumor microenvironment can also serve as a new therapeutic target. This review explores the mechanisms by which BCAAs metabolic reprogramming promotes cancer immune evasion and immune suppression. Additionally, it discusses the potential of targeting BCAA metabolism as a therapeutic strategy to inhibit tumor growth, enhance anti-tumor immune responses, and overcome drug resistance.

## 2 Metabolism of BCAAs in the body and metabolic reprogramming of BCAAs in tumors

Branched-chain amino acids (BCAAs), including leucine, valine, and isoleucine, can only be supplemented through diet and account for approximately 35% of essential amino acids in proteins and 18% of all amino acids. Under normal conditions, there is a dynamic balance between the intake and consumption of BCAAs (Blair et al., 2021). The most common dietary sources of BCAAs are high-fat dairy products, meat, and synthetic fitness supplements, making them important nutrients. Generally, supplementing BCAAs or a diet rich in BCAAs is beneficial for maintaining metabolic balance in the body. However, long-term elevated circulating BCAA levels (20%–50% higher than normal physiological concentrations) (Wang et al., 2011) have been associated with obesity, type 2 diabetes mellitus (T2DM), cardiovascular diseases, and certain tumors (White and Newgard, 2019; Siddik and Shin, 2019; Zheng et al., 2024).

**FIGURE 1:**
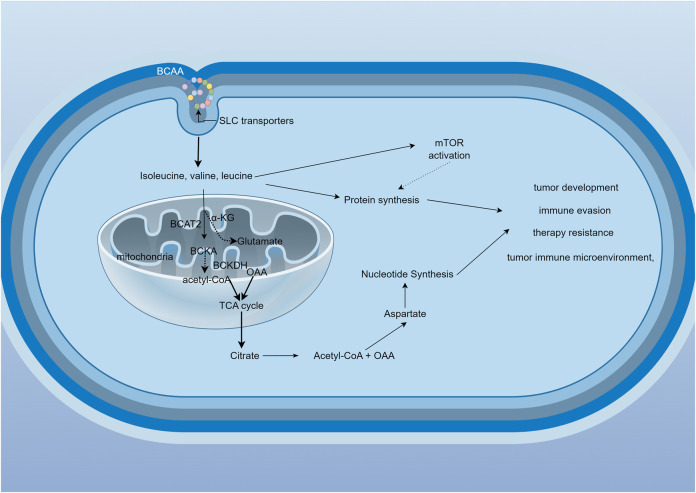
BCAAs metabolic cycle and related signaling pathways. BCAAs enter the cell *via* transporters and are metabolized by BCAT1 or BCAT2, generating branched-chain keto acids (BCKAs). These BCKAs are then processed by the BCKDH complex, which consists of three subunits: E1, E2, and E3. The catalytic efficiency of BCKDH depends on its phosphorylation status, regulated by BCKDK and PPM1K. BCKDK phosphorylates E1α to inhibit BCKDH, while PPM1K dephosphorylates the same site to activate BCKDH. Ultimately, acetyl-CoA is produced, entering the TCA cycle. Metabolites generated during this process may be associated with tumor development, immune evasion, therapeutic resistance, and the tumor microenvironment.

BCAAs are ingested through food (mainly from proteins) and are broken down by proteolytic enzymes in the gastrointestinal tract into individual amino acids, which are then absorbed into the bloodstream *via* the small intestine. Notably, gut microbiota, contribute approximately 12% of circulating BCAAs through proteolytic activity (Zhang et al., 2017; Pedersen et al., 2016). The gut microbiome, particularly *Prevotella copri* and *Bacteroides vulgatus*, promotes insulin resistance by increasing circulating branched-chain amino acids through enhanced microbial biosynthesis and reduced bacterial uptake (Pedersen et al., 2016). Once absorbed, BCAAs enter the bloodstream and are predominantly taken up by muscle tissue, which is rich in enzymes necessary for BCAA metabolism. Extracellular BCAAs utilize L-type amino acid transporters (LATs) to shuttle the cytoplasmic membrane into the cytoplasm (Peng et al., 2020) and the transport protein SLC25A44 assists BCAAs in entering mitochondria (Yoneshiro et al., 2019). These processes can all influence the levels of branched-chain amino acids (BCAAs) in plasma. The metabolism of BCAAs involves several steps (Figure 1): **Step 1:** In muscle and other tissues, BCAAs first undergo transamination catalyzed by branched-chain amino acid aminotransferase (BCAT), generating the corresponding branched-chain keto acids (BCKAs) (Dimou et al., 2022). This step converts BCAAs into α-keto acids, releasing amino groups that are used for amino acid synthesis or the urea cycle. **Step 2:** Oxidative decarboxylation occurs, where the generated branched-chain keto acids undergo further oxidative decarboxylation by the branched-chain keto acid dehydrogenase complex (BCKDH), producing their respective acyl-CoA derivatives (e.g., isovaleryl-CoA) (Peng et al., 2020). This process is the rate-limiting step of BCAA metabolism and mainly occurs in the liver and muscle (Du et al., 2022). **Step 3:** These acyl-CoA intermediates are further metabolized in the tricarboxylic acid (TCA) cycle, producing carbon dioxide, water, and energy. Leucine metabolism generates acetyl-CoA and acetoacetate, while isoleucine produces succinyl-CoA, and valine yields propionyl-CoA (Mann et al., 2021).

The metabolism of BCAAs is governed by multilayered regulatory networks involving enzymatic, hormonal, and microbial components. For instance, the activity of the BCKDH complex is regulated by phosphorylation and dephosphorylation (White et al., 2018). Changes in insulin and amino acid levels can also affect BCAA metabolism by modulating the activity of these key enzymes (Neinast et al., 2019). Tumor cells enhance BCAA synthesis through multiple pathways, including upregulating biosynthetic enzymes, metabolic reprogramming, nutrient scavenging, reductive carboxylation in hypoxia, and crosstalk with microenvironment. Studies have shown that elevated plasma BCAAs levels are associated with lung cancer and pancreatic cancer (Xu H. et al., 2023; Zhu et al., 2020; Katagiri et al., 2018). In these tumors, BCAA metabolism may be reprogrammed to fulfill the specific metabolic needs of tumor cells.

**FIGURE 2:**
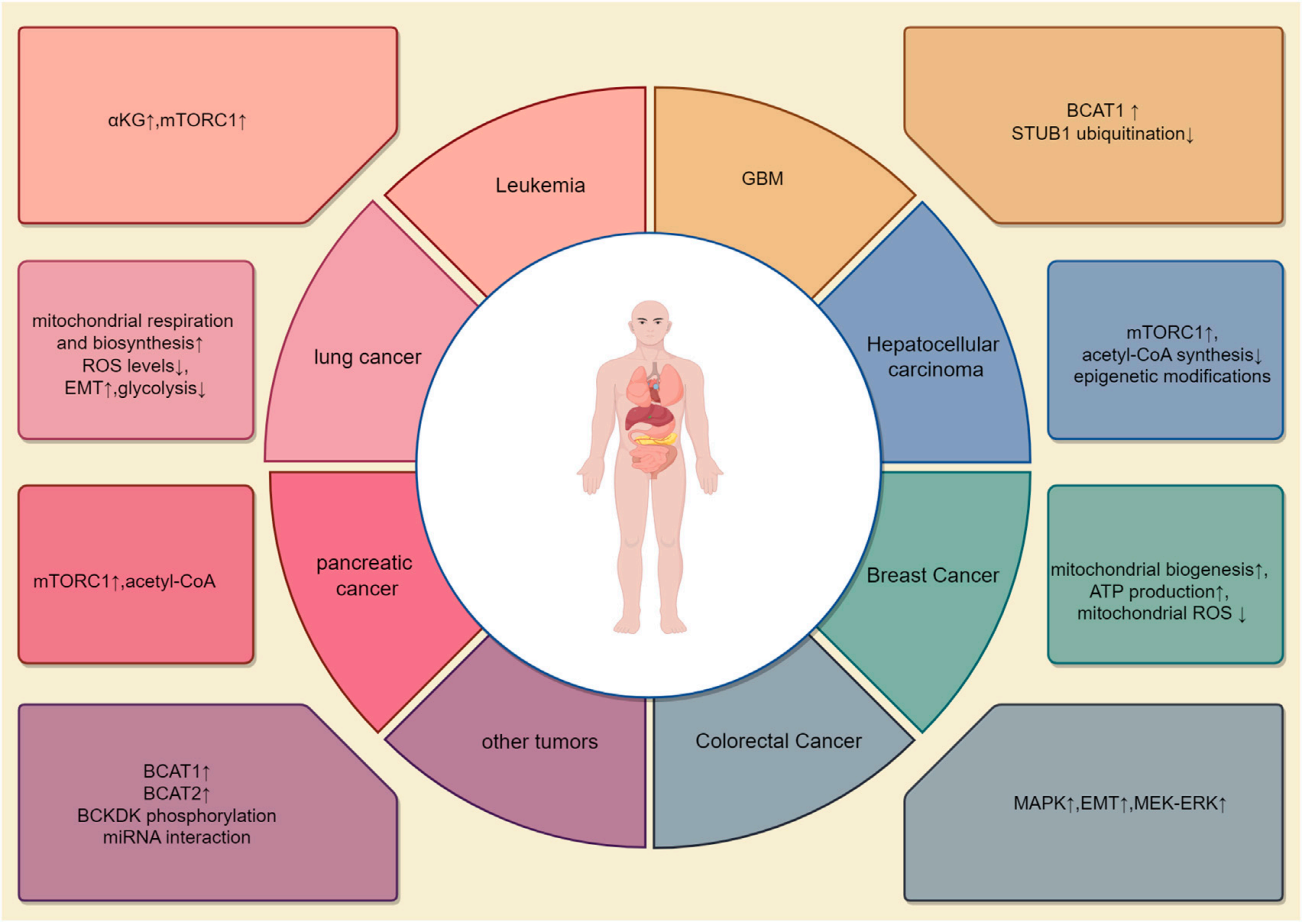
Metabolic reprogramming patterns in different tumors. The metabolism of BCAAs varies across different types of tumors. This article illustrates eight distinct BCAAs metabolic patterns, each with unique activated pathways, metabolic enzymes, and metabolic products. For example, in lung cancer, changes are seen primarily in BCAT1, while pancreatic cancer involves alterations in BCAT2. In colorectal cancer, metabolic changes focus on BCKDH-related enzymes. Additionally, BCAA metabolism in breast cancer is associated with reactive oxygen species (ROS) metabolism. BCAA metabolism interacts with other metabolic processes in a complex, interdependent manner.

The occurrence of different tumors is associated with distinct genetic backgrounds, and cellular studies have shown that specific gene mutations can promote diverse metabolic phenotypes (Garraway, 2013) (Table 1). However, it remains unclear whether the genetic background of the entire tumor tissue determines the metabolic pathways of different cancers. The metabolic differences between tumor types may also be attributed to cell-autonomous effects, with tumor metabolic gene expression being more similar to that of their tissue of origin compared to other tumors (Martínez-Jiménez et al., 2020). The same oncogenic drivers may also result in distinct metabolic phenotypes in lung and liver tumors (Yuneva et al., 2012). For example, in mouse and human tumor tissues, *Kras* activation and *Trp53* mutation deletion in the pancreas or lungs lead to pancreatic ductal adenocarcinoma (PDAC) or non-small-cell lung cancer (NSCLC). Although these tumors are caused by the same genetic mutations, they utilize BCAAs differently. NSCLC tumors incorporate free BCAAs into tissue proteins and use BCAAs as a nitrogen source, thereby increasing BCAAs uptake. In contrast, PDAC tumors show decreased BCAAs uptake. This suggests that the tissue of origin is a key determinant in how cancer meets its metabolic demands (Martínez-Jiménez et al., 2020). A striking paradox exists in pancreatic cancer: while obesity and diabetes (conditions with elevated blood BCAAs) increase PDAC risk, the tumors themselves show reduced BCAAs uptake. This likely occurs because PDAC cells downregulate BCAAs transporters and preferentially utilize other nutrients, leading to a disconnect between systemic BCAAs levels and tumor metabolic demands. The patterns of metabolic reprogramming of BCAAs are inconsistent across different tumors, with varying enzyme activities and pathway activations or inhibitions, resulting in significant metabolic alterations in various cancers (Figure 2).

**TABLE 1:** Patterns of BCAAS metabolic reprogramming in human cancers.

Cancer type	BCAAS intake	BCAT1	BCAT2	BCKDH	BCKDH	Other metabolic targets	Regulatory mechanism
lung cancer	↑	↑	↑	—	↑	—	mitochondrial respiration and biosynthesis↑, ROS levels↓, EMT↑, glycolysis↓
Hepatocellular carcinoma	↓	↑	↑	↑	↑	PPM1K↑, CPT1A↓, LAT1↑	mTORC1↑, acetyl-CoA synthesis↓, epigenetic modifications
pancreatic cancer	↓	↑	↑	—	↑	PPM1K↓, LAT1↑	mTORC1↑, acetyl-CoA
Colorectal Cancer	↑	—	↓	—	↑	*C.symbiosum*↑	MAPK↑, EMT↑, MEK-ERK↑
Leukemia	↑	↑	↑	↑	—	PPM1K↑, SLC7A5↑	αKG↑, mTORC1↑
Breast Cancer	↑	↑	—	↑	↑	LARS↓, LAT1↑	mitochondrial biogenesis↑, ATP production↑, mitochondrial ROS ↓

### 2.1 Lung cancer

In lung cancer, BCAAs metabolic reprogramming is significant. Tumor cells increase the uptake and metabolism of BCAAs to meet their rapid growth demands for energy and amino acids. The loss of enzymes responsible for BCAAs utilization, Bcat1 and Bcat2, impairs NSCLC tumor formation, although these enzymes are not essential for PDAC tumor formation (Li et al., 2020). BCAAs support the growth and proliferation of tumor cells by promoting the mTOR signaling pathway. Notably, studies have found that the plasma levels of BCAAs are typically elevated in lung cancer patients, indicating a close association between altered BCAA metabolism and tumor progression. BCAT1 can enhance BCAA metabolism, thereby increasing mitochondrial respiration and biosynthesis, reducing reactive oxygen species (ROS) levels, and ultimately enhancing NF-κB pathway signaling (Yu et al., 2022), promoting lung cancer development. High levels of BCAT1 promote the expression of SRY-box 2 (SOX2) by reducing alpha-ketoglutarate (α-KG), leading to the migration and metastasis of lung cancer cells (Mao et al., 2021). At the same time, BCAAs catabolism plays a crucial role in the metastasis of NSCLC cells, where the depletion of α-KG reduces the expression and activity of the m6A demethylase ALKBH5. As a result, the inhibition of ALKBH5 promotes the occurrence of epithelial-mesenchymal transition (EMT) in NSCLC cells and enhances their metastasis to the brain (Mao et al., 2024). Simultaneously, the upregulation of BCKDK affects the metabolism of BCAAs and citrate in NSCLC cells. Knockdown of BCKDK reduces NSCLC cell proliferation *in vitro* and induces apoptosis by inhibiting glycolysis while increasing oxidative phosphorylation and ROS levels, suggesting that BCKDK may promote NSCLC proliferation and could have clinical significance in treating NSCLC patients (Wang Y. et al., 2021). Rab1A, a small GTPase and an activator of mTORC1 as well as an oncogene, enhances Rab1A-mTORC1 signaling and promotes tumor proliferation by inhibiting BCAA catabolism in NSCLC, making it a potential biomarker for early diagnosis and identifying metabolism-based therapeutic targets in NSCLC patients (Xue et al., 2023). Additionally, studies suggest that SAR1B, a leucine sensor, influences cell growth through amino acid levels and controls mTOR complex 1 (mTORC1) by modulating mTORC1 signaling based on intracellular leucine levels. Selectively targeting SAR1B-dependent mTORC1 signaling could have potential for lung cancer treatment (Chen J. et al., 2021). Similarly, high doses of isoleucine can also inhibit the proliferation of lung cancer cells by stabilizing nuclear PTEN (Wang et al., 2023).

### 2.2 Hepatocellular carcinoma

In hepatocellular carcinoma (HCC), abnormal BCAA metabolism is also very common. Liver cancer cells often break down BCAAs to generate energy, enhance their antioxidant capacity, and promote tumor growth. BCAAs levels in liver cancer patients are often reduced (Marchesini et al., 2003), especially in the late stages of liver failure, suggesting that BCAAs could serve as potential therapeutic targets or biomarkers. In human hepatocellular carcinoma and liver cancer animal models, inhibition of BCAAs catabolic enzyme expression leads to BCAAs accumulation in tumors, and the degree of enzyme inhibition is closely associated with tumor aggressiveness (Ericksen et al., 2019), making it an independent predictor of clinical outcomes. Approximately 40 enzymes are involved in BCAA catabolism, and their transcripts are widely suppressed in liver tumors.

Studies have shown that in the absence of glutamine, BCAA catabolism is activated in cancer cells, enhancing BCAAs breakdown to stimulate cell proliferation and survival. PPM1K (Protein Phosphatase, Mg2^+^/Mn2^+^ Dependent 1K) is a mitochondrial serine/threonine phosphatase that plays a key role in regulating BCAA metabolism (Yang et al., 2022). It dephosphorylates and activates the branched-chain α-keto acid dehydrogenase complex (BCKD), promoting BCAA catabolism. Stabilizing PPM1K protein leads to enhanced BCAAs and BCKDHA degradation due to increased dephosphorylation. High expression of dephosphorylated BCKDHA and PPM1K promotes tumorigenesis, making BCKDHA and PPM1K potential therapeutic targets and predictive biomarkers for liver cancer. Additionally, BCAA metabolism is linked to lipid metabolism *via* carnitine palmitoyl transferase 1 (CPT1A), the rate-limiting enzyme of fatty acid oxidation (FAO). CPT1A is widely downregulated in liver tumor tissues and is associated with poor prognosis in HCC, promoting HCC progression in both new liver tumors and xenograft tumor models. This could be due to the disruption of acetyl-CoA synthesis, reducing histone acetylation and impairing BCAAS catabolism, leading to BCAAs accumulation and excessive mTOR signaling activation (Liu et al., 2024).

PROX1 expression is reduced by glucose starvation or AMPK activation and elevated in tumors with liver kinase B1 (LKB1) deficiency. Inhibiting PROX1 activation decreases BCAAs degradation by regulating epigenetic modifications and suppressing mTOR signaling (Paput et al., 2011). The LKB1-AMPK axis in cancer cells depends on PROX1 to maintain intracellular BCAAs pools. Cancer cells lacking the LKB1-AMPK axis rely on PROX1 to maintain intracellular BCAA levels, leading to enhanced mTOR signaling, tumorigenesis, and invasiveness.

LAT1 is a transmembrane amino acid transporter responsible for transporting large neutral amino acids such as leucine, isoleucine, valine, phenylalanine, and tyrosine from outside the cell to the inside. In liver cancer, it has been found that inhibiting LAT1 can reduce BCAAs transport activity and significantly lower cell proliferation (Kim et al., 2023). LAT1 ablation results in a significant reduction in phosphorylated p70S6K, with downstream mTORC1 signaling being suppressed. Therefore, inhibiting LAT1 activity may be an effective therapeutic strategy for liver cancer.

The role of BCAAs supplementation in liver cancer treatment has been explored in numerous studies, particularly in patients with liver cirrhosis and hepatocellular carcinoma (HCC) (van Dijk et al., 2023). BCAAs have a unique role in the nutritional intervention of liver diseases. Perioperative BCAA intake has been shown to decrease postoperative infections and ascites in liver cancer patients (Yap et al., 2023) and enhance survival in cirrhotic individuals (Hanai et al., 2020). BCAAs, particularly leucine, can activate the mTOR pathway, improving the function of immune cells such as T cells and natural killer cells, thereby boosting the anti-tumor immune response (Peng et al., 2020). While BCAAs supplementation has many potential benefits, there are also some controversies (Sideris et al., 2023). Some studies suggest that excessive BCAAs supplementation may promote the growth of certain tumor cells by activating the mTOR signaling pathway (Ericksen et al., 2019), thus requiring cautious use in liver cancer patients, especially with individualized adjustments based on the patient’s condition and nutritional needs. Although BCAAs supplementation is primarily used to support the nutrition and immune function of liver cancer patients, some research suggests that BCAAs may also affect tumor progression by inhibiting cancer cell proliferation and invasion. Certain BCAA metabolites may have inhibitory effects on cancer cells, particularly by modulating the mTOR signaling pathway and other metabolic pathways. However, the specific mechanisms involved still require further investigation. Additionally, studies have found that ferroptosis can regulate tumor metabolism and iron-dependent lipid peroxidation, thereby inhibiting tumor proliferation. Elevated BCAT2 expression in liver and pancreatic cancers is associated with reduced ferroptosis-related cell death. It has also been demonstrated that sorafenib and sulfasalazine have synergistic effects in inhibiting BCAT2 expression and inducing ferroptosis. Targeting BCAT2 may provide insights into overcoming resistance to sorafenib treatment (Wang K. et al., 2021).

### 2.3 Pancreatic cancer

Pancreatic hormone secretion is associated with obesity and insulin resistance. The development of pancreatic cancer can lead to insulin resistance and diabetes (Rossmeislová et al., 2021). However, the exact relationship between BCAA metabolism, PDAC progression, and tissue type remains unclear. Pancreatic cancer cells typically undergo metabolic reprogramming to meet the demands of rapid growth and proliferation. Studies have also found elevated levels of BCAAs in the blood of pancreatic cancer patients, strongly correlated to tumor progression. High levels of BCAA metabolism are linked to increased aggressiveness and poor prognosis in pancreatic cancer.

BCATs, including BCAT1 and BCAT2, transfer amino groups from BCAAs to α-KG. BCAT2 levels are higher in pancreatic cancer cell lines compared to normal cell lines (Li et al., 2020), making it a potential clinical target for pancreatic cancer therapy. BCAT2 is acetylated at lysine 44 (K44), an evolutionarily conserved residue. Acetylation of BCAT2 leads to its degradation through the ubiquitin-proteasome pathway and is stimulated during BCAAS deprivation. CREB-binding protein (CBP) and Sirtuin 4(SIRT4) are BCAT2’s acetyltransferase and deacetylase, respectively, controlling K44 acetylation in response to BCAAs availability (Lei et al., 2020). The K44R mutant enhances BCAAS catabolism, cell proliferation, and pancreatic tumor growth. This reveals a previously unknown regulatory mechanism of BCAT2 in PDAC and provides a potential therapeutic target for PDAC treatment.

Additionally, studies suggest that co-targeting stromal BCAT1 and the cancerous BCKDH complex impairs tumor cell proliferation and survival (Zhu et al., 2020). Cancer-associated fibroblasts (CAFs) take up extracellular matrix (ECM) components under nutrient-restricted conditions, with fibroblasts upregulating the Urokinase-type plasminogen activator receptor-associated protein (uPARAP) receptor for ECM uptake. CAFs can secrete ECM and induce a fibrotic environment within tumors. Enzymes or transporters related to BCAA metabolism, such as LAT1, may serve as potential therapeutic targets. Inhibiting BCAA metabolism can reduce tumor cells’ access to BCAAs, decreasing mTOR pathway activity, thereby inhibiting tumor growth and metastasis. A BCAAS-rich diet promotes pancreatic cancer development through USP1-mediated BCAT2 stabilization, and BCAAS intake is positively correlated with pancreatic cancer risk (Li J. T. et al., 2022; Rossi et al., 2022).

### 2.4 Colorectal cancer

In colorectal cancer (CRC), BCAA metabolic reprogramming enables tumor cells to adapt to nutritional stress in the microenvironment, enhancing their survival capacity. The accumulation of BCAAs caused by BCAT2 deficiency promotes chronic activation of mTORC1, mediating the carcinogenic effect of BCAAs (Kang Z. R. et al., 2024). BCKDK can also promote CRC development by upregulating the MEK-ERK signaling pathway. BCKDK is upregulated in CRC tissues, and increased BCKDK expression is associated with metastasis and poor clinical prognosis in CRC patients. Knockdown of BCKDK reduces CRC cell migration and invasion *in vitro* and lung metastasis *in vivo*. BCKDK promotes EMT by decreasing the expression of the epithelial marker E-cadherin and increasing the expression of mesenchymal markers N-cadherin and vimentin (Tian et al., 2020). Src phosphorylates BCKDK, enhancing its activity and stability, thereby promoting CRC cell migration, invasion, and EMT. Additionally, studies have shown that BCKDK enhances the MAPK signaling pathway by directly phosphorylating MEK, rather than through branched-chain amino acid catabolism, thereby promoting colorectal cancer progression (Xue et al., 2017). BCKDK may serve as a novel therapeutic target for colorectal cancer. BCAAs are also involved in maintaining redox balance, which plays an important role in the growth of colorectal cancer cells.

Recent studies have demonstrated that *C. symbiosum* selectively enriches in tumor tissues of colorectal cancer (CRC) patients and is associated with higher recurrence of colorectal adenomas after endoscopic polypectomy. The tumorigenic effect of *Clostridium symbiosum* has been observed in various mouse models (Ren et al., 2024). The mechanism involves *C. symbiosum* enhancing cellular cholesterol synthesis through BCAAs production, which in turn activates the Sonic Hedgehog signaling pathway. *C. symbiosum* has been identified as a bacterial driver of colorectal tumorigenesis, providing a potential target for CRC prediction, prevention, and treatment. Dietary supplementation with BCAAs may improve insulin resistance and inhibit the activation of the IGF/IGF-IR axis, thereby preventing the development of obesity-related colorectal cancer precursors. BCAAs may be an effective strategy for preventing colorectal cancer in obese individuals (Rossi et al., 2021). However, whether BCAAs intake affects the prognosis of colorectal cancer patients remains controversial (Shimizu et al., 2009; Long et al., 2021), and further research is needed to explore its role and mechanism in colorectal cancer.

### 2.5 Metabolic reprogramming of BCAAs in leukemia

Similar to many other tumor cells, leukemia cells reprogram the metabolism of BCAAs to meet the demands of rapid proliferation. Studies have shown that BCAAs are highly absorbed and quickly broken down in leukemia cells, providing energy and generating key metabolites such as nucleotides and lipids, which are essential for tumor cell proliferation (Neinast et al., 2019). In primary leukemia cells, BCAT1 actively breaks down BCAAs into branched-chain α-keto acids using α-KG, supplying key substrates for the tricarboxylic acid cycle and the synthesis of non-essential amino acids. Both processes help maintain α-KG levels, which are crucial for sustaining leukemia stem cell function (Kikushige et al., 2023). Research has found that BCAT1 is abnormally activated in chronic myeloid leukemia (CML) in both humans and mice, promoting BCAAs production *via* the MSI2-BCAT1 axis, thereby driving the development of myeloid leukemia (Hattori et al., 2017). Moreover, studies have shown that BCAT1 knockout leads to an accumulation of α-KG, which is a vital cofactor for α-KG-dependent dioxygenases, such as the Egl-9 family hypoxia-inducible factor 1 (EGLN1) and the ten-eleven translocation (TET) family of DNA demethylases. This results in EGLN1-mediated degradation of HIF1α, suppressing tumor cell proliferation (Raffel et al., 2017). In AML cells with high levels of BCAT1, a DNA hypermethylation phenotype similar to cases with mutant isocitrate dehydrogenase (IDHmut) has been observed, and this is associated with poor disease prognosis.

Several genes associated with poor leukemia prognosis are also linked to BCAA metabolic pathways. EZH1, a homolog of EZH2, is essential for the initiation of leukemia in EZH2-deficient cells and contributes to epigenetic vulnerability. EZH2 inactivation leads to BCAT1 overactivation, enhancing BCAA metabolism and mTOR signaling, which together drive the transformation of myeloproliferative neoplasms into leukemia (Gu et al., 2019). METTL16, an m6A methyltransferase and one of the most common internal modifiers of mammalian mRNAs, is abnormally overexpressed in human AML cells. Through an m6A-dependent mechanism, METTL16 promotes the expression of BCAT1 and BCAT2, reprogramming BCAA metabolism in AML and contributing to leukemogenesis (Han et al., 2023). GPRC5C, a member of the G protein-coupled receptor family group C, is a regulator of hematopoietic stem cell dormancy and is associated with poor leukemia prognosis. Elevated intracellular BCAAs levels, a tumor metabolic characteristic, are reversed after Gprc5c depletion. Targeting the BCAAs transporter SLC7A5 with JPH203 inhibited oxidative phosphorylation and exerted anti-leukemic effects, suggesting that the GPRC5C-SLC7A5-BCAAs axis may serve as a therapeutic target (Zhang Y. W. et al., 2023).

### 2.6 Breast cancer

In breast cancer, BCAA metabolism is also related to tumor occurrence and progression. Breast cancer cells maintain their rapid proliferation rate by increasing the uptake and utilization of BCAAs. Elevated expression of BCAT1 has been observed in breast cancer, and knocking down BCAT1 can inhibit the growth and proliferative capacity of breast cancer cells (Zhang and Han, 2017). BCAT1 promotes mitochondrial biogenesis, ATP production, and inhibits mitochondrial ROS in breast cancer cells by regulating the expression of related genes. High concentrations of BCAAs affect the migration and invasion capabilities of breast cancer cells. Elevated BCAAs inhibit tumor metastasis and cell invasion abilities and reduce the expression of N-cadherin, indicating that high BCAAs levels may suppress breast cancer tumor growth and metastasis (Tobias et al., 2021). This suggests that a high-BCAAs diet could have potential therapeutic significance in breast cancer treatment (Chi et al., 2022). Leucyl-tRNA synthetase (LARS) is inhibited in breast cell transformation and human breast cancer (Stine et al., 2022). *In vitro* experiments demonstrated that inhibition of BCKDK expression reduced the migration of human breast cancer cells, while *in vivo* it decreased lung metastasis. BCKDK inhibited the interaction between talin1 and the E3 ubiquitin ligase TRIM21, leading to reduced ubiquitination and degradation of talin1, thereby suppressing tumor cell migration (Xu C. et al., 2023). This study found that LAT1, a key amino acid transporter, plays a role in AI-resistant breast cancer by promoting leucine uptake and mTORC1 signaling. LAT1 expression increased in resistant tumors and was linked to advanced stages. The LAT1 inhibitor JPH203 reduced cell proliferation in resistant cells, suggesting LAT1 as a potential therapeutic target in AI-resistant breast cancer (Shindo et al., 2021). Additionally, studies have shown that elevated circulating BCAAs levels are associated with a reduced risk of breast cancer in premenopausal NHSII women but an increased risk in postmenopausal NHS women (Zeleznik et al., 2021).

### 2.7 Other tumor types

Most malignant tumors exhibit elevated levels of BCAT1, which is associated with malignant phenotypes in various cancers, such as nasopharyngeal carcinoma (NPC) (Zhou et al., 2013), gastric cancer (Qian et al., 2023), melanoma (Mao et al., 2021), and astrocytoma (Tönjes et al., 2013), as well as poor prognosis in cancer. The promoter encoding BCAT1 can interact with RNA-binding motif proteins, promoting tumorigenesis in nasopharyngeal carcinoma (Xu X. C. et al., 2021). The long non-coding RNA GAS6-AS2 has been identified as a key tumor growth driver in osteosarcoma (Wei et al., 2020) by inhibiting miR-934. Solid evidence from various cancers has demonstrated BCAT1’s direct regulation in the mTOR pathway. BCAT1-mediated mTOR activation is involved in the lethal biological behaviors of gastric cancer (Shu et al., 2021)and cervical cancer (Luo et al., 2021). Similarly, the regulation of BCAT2 expression in cancer has been reported in the literature. Recent studies have shown that compared to normal tissues, BCAT2 expression is elevated in cancers such as bladder cancer (Cai et al., 2023), pancreatic cancer (Li et al., 2020; Lee et al., 2019), breast cancer (Zhang and Han, 2017), and non-small cell lung cancer (NSCLC) (Lee et al., 2019). The study found that BCAT1 is phosphorylated by BCKDK in glioblastoma, which enhances its activity and stability while inhibiting its degradation mediated by STUB1 ubiquitination, thereby promoting tumor growth. Inhibiting the BCKDK-BCAT1 axis can increase sensitivity to temozolomide (TMZ), suggesting this pathway as a potential therapeutic target (Wang W. et al., 2024).

Overexpression of BCKDH protein levels has been observed during carcinogenesis in most ovarian cancer cell lines (Ibrahim et al., 2023), oral squamous cell carcinoma (Grimm et al., 2016), osteosarcoma (Zhang and Han, 2017), and melanoma (Tian et al., 2023). Active BCKDH is tightly regulated by its phosphorylation status, which is determined by BCKDK and PPM1K levels. High expression of BCKDK and certain malignant proliferation behaviors have been confirmed in colorectal cancer (Lei et al., 2020), breast cancer (Xu C. et al., 2023), and NSCLC (Xue et al., 2023).

## 3 Research on mechanisms of BCAAs-induced tumor resistance

Tumor resistance refers to the phenomenon where tumor cells develop resistance to cancer therapies, rendering previously effective treatments ineffective or significantly less effective. This resistance can either be intrinsic (i.e., primary resistance) or acquired over the course of treatment (i.e., acquired resistance) (Bagchi et al., 2021).Tumor resistance typically involves a variety of complex biological mechanisms, including genetic mutations, activation of signaling pathways, drug efflux, enhanced DNA repair, and evasion of apoptosis (Vesely et al., 2022). The development of resistance makes tumors harder to control and treat, presenting a major challenge in cancer therapy.

**FIGURE 3:**
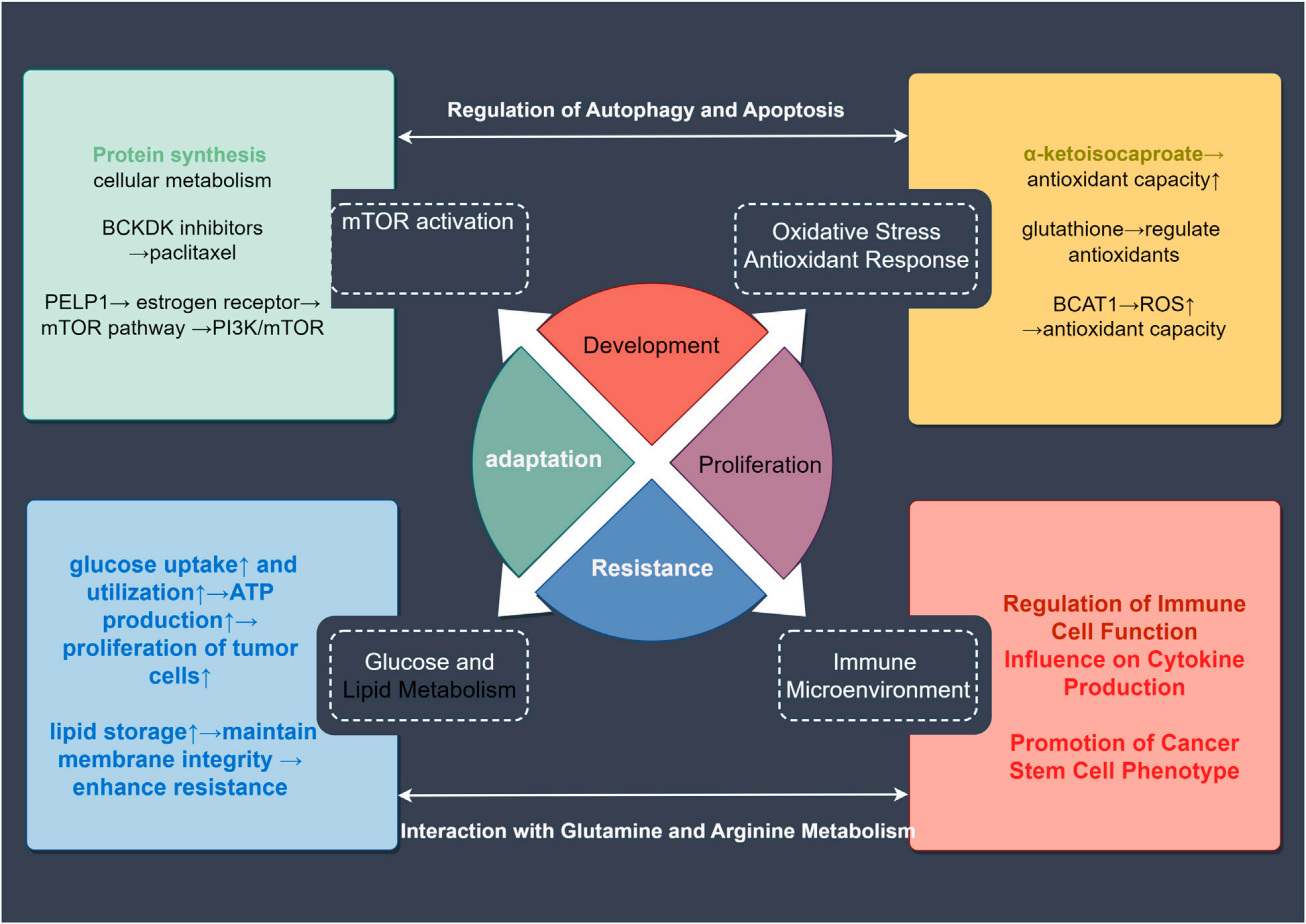
Mechanisms of BCAA-Related tumor drug resistance. Abnormal BCAA metabolism can lead to resistance in tumor-related therapies. This article covers four major aspects of this resistance: activation of the mTOR signaling pathway, oxidative stress response, alterations in glucose and lipid metabolism, and changes in the tumor microenvironment. These factors can further interact through mechanisms such as autophagy, apoptosis, and metabolic changes to enhance tumor resistance. These insights provide valuable reference points for targeting resistance in treatment.

BCAAs induce tumor resistance through multiple mechanisms. These include activation of the mTOR signaling pathway to promote tumor cell growth, regulation of oxidative stress responses to resist oxidative damage induced by treatment, modulation of glucose and lipid metabolism to support tumor cells’ adaptation to changing energy demands, and shaping the immune microenvironment to suppress anti-tumor immune responses (Figure 3). Additionally, BCAAs regulate autophagy and apoptotic pathways, preventing therapy-induced cell death, thereby enhancing tumor cell survival. Collectively, these mechanisms contribute to the development of treatment resistance in tumor cells.

### 3.1 Activation of the mTOR signaling pathway

BCAAs, particularly leucine, activate the mTORC1 (mammalian target of rapamycin complex 1) pathway by directly binding to it. Persistent activation of mTOR signaling is closely linked to tumor growth, proliferation, and survival in many types of cancer (Zhan et al., 2023). By enhancing the activity of this pathway, BCAAs help tumor cells maintain survival under the stress of anticancer drugs. For instance, mTOR signaling activation can counteract the growth-inhibiting effects of chemotherapy by promoting protein synthesis and cellular metabolism. In many cancers, mTOR inhibitors have been considered as potential therapeutic drugs, but the activation of mTOR signaling by BCAAs may lead to drug resistance (Liu et al., 2022). In some cancer types, inhibiting the mTOR signaling pathway is thought to enhance drug sensitivity, suggesting that abnormal BCAA metabolism may promote resistance (Bansal and Simon, 2018).

For example, BCKDK inhibitors can disrupt the mTORC1-Aurora axis, thereby enhancing the sensitivity of breast and ovarian cancer cells to chemotherapeutic drugs. The use of BCKDK inhibitors can reverse the cell cycle arrest induced by paclitaxel. BCKDK might play an important role in increasing the sensitivity of tumor cells to paclitaxel. Certain breast cancer cells reduce sensitivity to PI3K/mTOR inhibitors by enhancing mTOR pathway activity through leucine metabolism (Ibrahim et al., 2023). Proline, Glutamate, Leucine-Rich Protein 1 (PELP1), a proto-oncogene that regulates estrogen receptor (ER) signaling, interacts with serine/threonine protein kinase mTOR and modulates mTOR signaling (Gonugunta et al., 2014). mTOR inhibitors can sensitize PELP1-expressing cells to hormone therapy.

### 3.2 Oxidative stress and antioxidant response

Alterations in BCAA metabolism can affect tumor cells’ responses to oxidative stress. Oxidative stress is often a key cytotoxic mechanism in chemotherapy and radiotherapy, inducing oxidative damage that leads to tumor cell death. However, products of BCAA metabolism, such as α-ketoisocaproate (produced from leucine breakdown), can enhance the antioxidant capacity of tumor cells, helping them resist the oxidative damage caused by chemotherapy and radiotherapy (Cai et al., 2022). This mechanism allows tumor cells to alleviate oxidative stress by regulating antioxidants such as glutathione, further promoting drug resistance (Zhang B. et al., 2022). These data suggest that high BCAAs concentrations may have deleterious effects on circulating blood cells, contributing to the pro-inflammatory and oxidative states observed under several pathophysiological conditions (Zhenyukh et al., 2017). Hypoxia-inducible factors (HIFs) regulate metabolic reprogramming in response to hypoxia. LAT1 is a transporter of BCAAs, and studies have found that hypoxia upregulates the mRNA and protein levels of LAT1 and BCAT1 in human glioblastoma (GBM) cell lines through the binding of HIF-1α and HIF-2α to the intron of the BCAT1 gene. However, hypoxia does not upregulate their homologs LAT2-4 and BCAT2. This allows tumor cells to continue proliferating under hypoxic conditions (Zhang et al., 2021). Moreover, studies indicate that enhanced BCAA metabolism boosts the activity of antioxidant enzymes, such as glutathione, helping tumor cells resist treatment-related accumulation of reactive oxygen species (ROS) (Pavlova et al., 2022). Studies have found that BCAT1 may possess stronger antioxidant properties compared to BCAT2. The BCAT1-CXC motif has a novel antioxidant function, with this CXXC motif proven to act as a “redox switch” in the enzymatic regulation of BCAT proteins. The BCAT1-CXC motif may help buffer ROS levels within AML cells, influencing cell proliferation, which could impact the ROS-mediated development of myeloid leukemia (Hillier et al., 2022). Low-grade gliomas and secondary glioblastomas lead to excessive production of (R)-2HG, which can effectively inhibit 2OG-dependent transaminases BCAT1 and BCAT2. By reducing glutamate levels, this inhibition sensitizes IDH-mutant glioma cells specifically to glutaminase, making them more susceptible to oxidative stress *in vitro* and to radiation both *in vivo* and *in vitro* (McBrayer et al., 2018). Chemotherapeutic agents kill tumor cells by inducing oxidative stress, and the regulation of BCAA metabolism may enhance drug resistance by mitigating this stress (Pavlova and Thompson, 2016). The C-terminal of Hsc70-interacting protein (CHIP) is an E3 ubiquitin ligase, and its coiled-coil (CC) domain interacts with BCAT1. Through the CHIP/BCAT1 axis, it enhances glioma sensitivity to temozolomide by reducing glutathione (GSH) synthesis and increasing oxidative stress (Lu et al., 2024a).

### 3.3 Interaction with glucose and lipid metabolism

The cross-regulation between BCAA metabolism and glucose and lipid metabolism plays a crucial role in metabolic reprogramming within tumor cells. Tumor cells often adapt to nutrient limitations and anticancer drug pressure by readjusting metabolic pathways. BCAA metabolism promotes glucose uptake and utilization, increasing ATP production, thereby helping tumor cells maintain energy supply and cope with drug pressure (Fang et al., 2022). Moreover, the interaction between BCAAs and lipid metabolism can support the rapid growth and repair of cell membranes by providing precursors for lipid synthesis, which is essential for tumor cell survival. Studies have shown that BCAAs enhance glucose uptake, increase glycolytic products, and promote tumor cell survival in harsh environments by activating the PI3K/AKT pathway (Zoncu et al., 2011). Alterations in lipid metabolism, such as increased lipid storage promoted by BCAAs, help tumor cells maintain membrane integrity and enhance resistance to chemotherapy (Bacci et al., 2021). Osimertinib, a third-generation EGFR tyrosine kinase inhibitor (TKI), has shown significant clinical efficacy in treating non-small cell lung cancer (NSCLC). Studies have found that in TKI-resistant cells, upregulated BCAT1 reprograms BCAA metabolism and promotes α-ketoglutarate (α-KG)-dependent demethylation of histone H3 at lysine 27 (H3K27), leading to the de-repression of glycolysis-related genes, thereby enhancing glycolysis and promoting tumor progression. WQQ-345, a novel BCAT1 inhibitor, has demonstrated antitumor activity in both *in vitro* and *in vivo* models of TKI-resistant lung cancer with high BCAT1 expression. BCAT1 is a promising target for treating TKI-resistant NSCLC (Zhang T. et al., 2024). PPM1K regulates glycolysis to generate hematopoietic stem cells and leukocytes through the ubiquitination of MEIS1 and p21 mediated by CDC20. Inhibition of PPM1K extended the survival time of mice in leukemia models, suggesting that PPM1K could be used in combination with chemotherapy drugs for leukemia to improve treatment efficacy (Liu et al., 2018).

### 3.4 Impact on the immune microenvironment

The immunosuppressive nature of the tumor microenvironment plays a crucial role in tumor drug resistance. BCAA metabolism regulates immune responses by affecting the metabolic activity of immune cells. Research shows that T cells and natural killer (NK) cells require an adequate supply of BCAAs to maintain their antitumor functions. When BCAA metabolism is disrupted, the activity of these immune cells may be suppressed, leading to enhanced immunosuppression within the tumor microenvironment. Immunotherapy relies on the host immune system’s ability to recognize and eliminate cancer cells, but changes in BCAA metabolism may weaken this effect, promoting immune evasion by tumor cells.

#### 3.4.1 Regulation of immune cell function

BCAA metabolism influences the activity and function of key immune cells, including T cells, macrophages, myeloid-derived suppressor cells (MDSCs), and NK cells. Pan-cancer biological analyses show that the infiltration levels of CD4^+^ T cells, CD8^+^ T cells, B cells, neutrophils, and macrophages in lung cancer, colorectal cancer, and head and neck squamous cell carcinoma are correlated with the expression of BCAT1 (Li G. S. et al., 2022). These immune cells jointly regulate the immune tumor microenvironment and play critical roles in immunotherapy. BCAA metabolism exhibits dual immunomodulatory roles in the tumor microenvironment (TME), with both pro-tumoral and anti-tumoral effects on key immune cells.

##### 3.4.1.1 T cells

BCAAs exhibit dual roles in T cell activation, proliferation, and differentiation. In promoting tumor progression, BCAAs depletion in the tumor microenvironment can inhibit T cell function, reducing their ability to mount an effective antitumor immune response (Xia et al., 2021). While enhanced BCAA metabolism may promote the survival and function of regulatory T cells (Tregs), which suppress immune responses and help tumors evade immune detection (Ikeda et al., 2017). In suppressing tumor progression, BCAAs, particularly leucine, are indispensable amino acids for immune regulation through metabolic reprogramming. However, the molecular mechanisms underlying this phenomenon remain unclear. Many studies have shown that solute carrier (SLC) transporters play new roles in the tumor microenvironment by altering immune cell metabolism (Chen and Chen, 2022; Nachef et al., 2021; Kocher et al., 2021). SLC1A5, SLC7A5, and SLC3A2 are the most highly expressed genes encoding amino acid transport proteins in the tumor microenvironment (O’Sullivan et al., 2019). The most abundant amino acid transporter in activated T cells is SLC7A5 (Kanai, 2022; Meng et al., 2024). Studies have found that T cell receptor (TCR) activation increases the expression of BCAT1 and SLC7A5 in human CD4^+^ T cells, promoting leucine influx and catabolism, which is particularly important for the T helper cell (Th17) response. Inhibiting SLC transporters reduces the ability of immune cells to eliminate tumor cells. SLC7A5 is involved in T cell differentiation, activation of the mTORC1 signaling pathway, and c-Myc expression, while knocking out SLC3A2 prevents T cell expansion (Ikeda et al., 2017; Najumudeen et al., 2021; Zhang C. et al., 2024). Chimeric antigen receptor (CAR)-T cells are an innovative immunotherapy where T cells are genetically engineered. Research has shown that traditional T cells or CAR-T cells can compete with tumor cells for amino acids. Artificially increasing the expression of SLC7A5 or SLC7A11 transmembrane amino acid transporters has been shown to enhance CAR-T cell proliferation and antitumor activity by upregulating intracellular arginase (Panetti et al., 2023). Additionally, BCKDK-engineered CAR T cells were designed to reprogram BCAA metabolism in the tumor microenvironment based on genotype and phenotype modifications, enhancing the ability of T cells to eliminate cancer cells (Yang et al., 2024). In an experimental autoimmune encephalomyelitis (EAE) model, blocking BCAT1-mediated leucine catabolism using BCAT1 inhibitors or LβhL treatment alleviated the severity of EAE by reducing HIF1α expression and IL-17 production in spinal cord mononuclear cells. Activated CD4^+^ T cells induce an alternative pathway of cytosolic leucine catabolism through BCAT1 and hydroxyphenylpyruvate dioxygenase (HPD)/HPDL, producing the key metabolite β-hydroxy-β-methylbutyrate (HMB). HMB helps regulate the mTORC1-HIF1α pathway by increasing HIF1α mRNA expression, a major signaling pathway for IL-17 production. Treatment with L-β-hydroxyisoleucine (LβhL), a leucine analog and competitive inhibitor of BCAT1, can reduce IL-17 production in TCR-activated CD4^+^ T cells, thus weakening the immune response in the tumor microenvironment (Kang Y. J. et al., 2024).

Immune checkpoint inhibitors (ICIs) have improved survival rates in patients with advanced cancer (such as bladder cancer, BLCA). However, their overall efficacy remains limited, as many patients still develop resistance to immunotherapy. Recent studies have found that LRFN2 forms a non-inflammatory tumor microenvironment (TME) in BLCA. Tumor-intrinsic leucine-rich repeat and fibronectin type III domain-containing protein LRFN2 suppresses the recruitment and functional transformation of CD8^+^ T cells by reducing the secretion of pro-inflammatory cytokines and chemokines. LRFN2 inhibits antitumor immunity by reducing CD8^+^ T cell infiltration, proliferation, and differentiation *in vitro*. Furthermore, spatially exclusive relationships between LRFN2^+^ tumor cells and CD8^+^ T cells, as well as markers such as programmed cell death-1 (PD-1) and T cell factor 1 (TCF-1), have been observed, thereby enhancing tumor resistance (Yu et al., 2023). Additionally, BCAAs promote the effector function of CD8^+^ T cells and antitumor immunity by reprogramming glucose metabolism, which can enhance the clinical efficacy of anti-PD-1 immunotherapy against tumors (Yao et al., 2023).

##### 3.4.1.2 Macrophages

BCAA metabolism can regulate the polarization of macrophages. A BCAAS-enriched environment may inhibit M1 macrophages, which have antitumor functions, while promoting M2 macrophages (TAM 2), which support tumor growth and immune suppression. TAM 2 infiltration is significantly elevated in the pancreatic tumor microenvironment (CME). (Zhang et al., 2023b). Increased levels of TAM two drive the tumor-promoting characteristics of cancer cells and are associated with poor disease prognosis. BCAT1, along with bone marrow stromal antigen 2 (BST 2) and the tyrosine kinase MERTK, promotes cancer progression by regulating TAM 2 polarization, offering a potential target for pancreatic cancer treatment. Another FN1-induced transcriptome network mediates immune cell infiltration in the CME of oral squamous cell carcinoma (Peng et al., 2023). Additionally, TAMs can be reprogrammed through diet or genetic modification to overcome MYC-overexpressing cancer cells *via* non-canonical phagocytosis-mediated, Rag GTPase-independent mTORC1 signaling (Zhang et al., 2023c).The regulatory role of BCAT1 in macrophage function holds therapeutic significance for inflammatory diseases. While BCAT1’s role in regulating macrophage function helps reduce the infiltration of inflammatory factors and has therapeutic potential for various inflammatory diseases (Papathanassiu et al., 2017), it is still unclear whether similar mechanisms exist in the TME.

##### 3.4.1.3 NK cells

Low concentrations of arginine can inhibit T cell proliferation and activity in the tumor microenvironment (TME), but increased expression of SLC7A5 can help NK cells in acute myeloid leukemia (AML) maintain their proliferative and activated phenotype under low arginine conditions, leading to AML cell apoptosis (Stavrou et al., 2023). In contrast, inhibiting SLC7A5 in cytokine-activated NK cells reduces c-Myc protein levels and mTORC1 signaling, thereby enhancing their antitumor effects (Loftus et al., 2018).

##### 3.4.1.4 MDSCs

BCAAs support the immunosuppressive activity of myeloid-derived suppressor cells (MDSCs), inhibiting antitumor immune responses and promoting tumor progression. A high-fat diet (HFD) is a high-risk factor that disrupts the gut microbiome, leading to the malignant progression of cancer. Both obesity and obesity-associated gut microbiota are linked to poor prognosis and advanced cachexia in female cancer patients. The HFD-related microbiota promotes cancer progression by generating polymorphonuclear myeloid-derived suppressor cells (PMN-MDSCs). The HFD microbiota releases an abundance of leucine, activating the mTORC1 signaling pathway in myeloid progenitor cells, thus promoting PMN-MDSC differentiation (Chen et al., 2024). Clinically, elevated leucine levels in the peripheral blood of female cancer patients, induced by the HFD microbiota, are associated with extensive tumor PMN-MDSC infiltration and poor clinical outcomes. BCAAs also affect the immunoregulatory properties of mesenchymal stem cells (MSCs). They regulate the S, G2, and M phases of the cell cycle, promoting MSC proliferation and metabolic activity (Zhang F. et al., 2022). In addition, in immune-related diseases, BCAAs modulate the immunoregulatory capacity of MSCs by increasing phosphorylated signal transducer and activator of transcription 3 (p-STAT3)/STAT3 signaling, reducing p-NF-κB/NF-κB signaling, and enhancing the production of anti-inflammatory TGF-β and prostaglandin E (Sartori et al., 2020).

#### 3.4.2 Influence on cytokine production

BCAA metabolism can affect the production of pro-inflammatory and anti-inflammatory cytokines in the tumor immune microenvironment. For example, BCAAs can activate the NF-κB pathway (Sartori et al., 2020), leading to the production of cytokines such as interleukin-6 (IL-6) and tumor necrosis factor-alpha (TNF-α), which can support tumor progression and alter immune responses. The expression of chemokines critical for CD8^+^ T cell recruitment, such as CCL3, CCL4, CCL5, CXCL9, and CXCL10, is hindered by BCAT2. Chemotaxis experiments show that BCAT2 is negatively correlated with CD8^+^ T cell cytotoxic INF-γ and TNF-α. More importantly, the loss of BCAT2 enhances the effectiveness of anti-PD-1 therapy (Cai et al., 2023).

### 3.5 Interaction with glutamine and arginine metabolism

BCAA metabolism intersects with glutamine metabolism, which is crucial for the function of rapidly proliferating cells, including tumor cells and certain immune cells. By influencing the availability and utilization of glutamine, BCAAs can regulate the immune microenvironment, affecting the balance between antitumor immune responses and immune suppression (Bader et al., 2020). Numerous studies have shown that the uptake of glutamine, arginine, and BCAAs is upregulated across various cancers and activates Th1 and CD8^+^ T cells (Chen C. L. et al., 2021). For example, in ovarian cancer cells, CD4^+^ and CD8^+^ memory T cells and M0 macrophages overexpress the arginine transporter CAT1. Silencing CAT1 transporter results in decreased BCAAS levels. Arginine acts as a crucial precursor for polyamine biosynthesis, and targeting key metabolic enzymes like arginase-1 (Arg1) can effectively regulate polyamine production in the tumor microenvironment (TME) (Wetzel et al., 2023). These polyamines possess well-documented immunosuppressive properties that promote tumor growth by inhibiting cytotoxic immune responses (Lian et al., 2022). Importantly, dendritic cells frequently overexpress Arg1, establishing it as a novel metabolic checkpoint within the TME (Martí and Reith, 2021). Through Arg1-mediated arginine depletion, dendritic cells may contribute to T cell exhaustion, a key factor in tumor immune evasion and immunotherapy resistance (Lian et al., 2022). Moreover, all three of these amino acids maintain cell growth and proliferation by activating mTORC1 in tumor and immune cells (You et al., 2022). mTOR signaling is dysregulated in cancer cells, whereas T cell function requires mTOR upregulation (Kim and Guan, 2019; Waickman and Powell, 2012). mTOR sensing may occur through Rag GTPase-dependent mechanisms and can interact with various protein targets (Bodineau et al., 2022). Glutamine, along with asparagine, activates mTOR signaling *via* a Rag-GTPase-independent mechanism (Meng et al., 2020). Leucine-driven mTOR activation involves SAR1B, GATOR1-2, and Sestrin2 (Chen J. et al., 2021; Saxton et al., 2016). Under low glutamine conditions, targeting ASCT2 renders breast cancer cells more sensitive to leucine uptake inhibition, suggesting that cancer cells with reduced transporter plasticity are more vulnerable to disruptions in amino acid homeostasis (Bröer et al., 2019). In conclusion, cancer and immune cells are influenced by arginine, glutamine, and BCAAs, and these three exist in a mutually balanced and regulated relationship. Understanding their interactions may provide new therapeutic targets for the treatment of the tumor immune microenvironment.

### 3.6 Regulation of autophagy and apoptosis

BCAA metabolism also helps tumor cells resist therapeutic pressure by influencing autophagy and apoptosis pathways. Autophagy is a survival mechanism that cells use during nutrient deprivation or stress, maintaining energy balance by degrading damaged organelles or proteins (Debnath et al., 2023). BCAA metabolism inhibits autophagy through the mTOR signaling pathway, thereby supporting the metabolic needs of tumor cells. Additionally, BCAAs metabolic products can regulate apoptosis signaling pathways, inhibiting chemotherapy-induced programmed cell death, leading to treatment resistance in tumor cells. BCKDK inhibitors suppress protein translation, impair mitochondrial function, accelerate apoptosis, and enhance the cytotoxicity of doxorubicin in triple-negative breast cancer (Biswas et al., 2021). Inhibition of BCAA metabolism promotes glioblastoma cell apoptosis by disrupting mitochondrial dynamics mediated by mitofusin 2 (Mfn2) and inhibiting the PI3K/AKT/mTOR pathway, making it a potential novel therapeutic target for treating glioblastoma (Lu et al., 2024b). In AML, BCAT1 affects cell proliferation and regulates the cell cycle, apoptosis, and DNA damage/repair processes. BCAT1 modulates histone methylation by reducing intracellular αKG levels in AML cells. High expression of BCAT1 enhances the sensitivity of AML cells to poly (ADP-ribose) polymerase (PARP) inhibitors both *in vivo* and *in vitro*. The increased sensitivity of high-BCAT1 AML to PARP inhibitors could serve as an effective therapeutic strategy for AML patients (Pan et al., 2024). Furthermore, BCAT1 is overexpressed following NOTCH1-induced leukemic progenitor transformation and controls BCAT1 expression by binding to the BCAT1 promoter. Depletion or inhibition of BCAT1 leads to the production of 3-hydroxybutyrate (3-HB), an endogenous histone deacetylase inhibitor, and is associated with increased sensitivity to DNA-damaging agents. The combined action of BCAT1 inhibition and etoposide can selectively eliminate tumors in human xenograft models, suggesting that BCAT1 inhibitors may play an important role in the treatment of refractory T-ALL (Tosello et al., 2024). The key transcription factor regulating autophagy, EB (TFEB), promotes the proliferation and metastasis of pancreatic cancer cells. Knockdown of TFEB inhibits PCC proliferation and metastasis by regulating BCAAS catabolism through BCAT1LAI. BCAAS deprivation, combined with the TFEB-targeting drug elthrombopag, can exert a dual effect by blocking both exogenous supply and endogenous utilization (Wang T. et al., 2024). Additionally, BCAAs suppress insulin-induced cancer cell proliferation by inducing autophagy (Wubetu et al., 2014).

### 3.7 Promotion of cancer stem cell phenotype

BCAA metabolism is associated with the maintenance and enhancement of cancer stem cells (CSCs). CSCs are a highly drug-resistant subpopulation of tumor cells. They typically exhibit significant metabolic plasticity, including enhanced BCAAs catabolism, enabling them to survive under adverse conditions such as chemotherapy or radiotherapy (Ma et al., 2018). By supporting the survival of CSCs, BCAA metabolism contributes to tumor recurrence and treatment resistance. In breast cancer studies, interferon-γ (IFNγ) produced by activated T cells has been shown to directly convert non-CSCs into CSCs. BCAT1 was identified as a downstream mediator of IFNγ-induced CSC plasticity, potentially contributing to immune checkpoint blockade (ICB) failure. Targeting BCAT1 has been demonstrated to improve cancer vaccination and immune checkpoint blockade by preventing IFNγ-induced CSC modification (Ma et al., 2018). In hepatocellular carcinoma (HCC) cells expressing the liver CSC marker EpCAM, inhibition of mTOR complex 2 (mTORC2) or activation of mTORC1 leads to reduced EpCAM expression, thereby decreasing the tumorigenic potential of CSCs and increasing sensitivity to the antiproliferative effects of 5-FU. BCAAs may reduce the number of CSCs through the mTOR pathway, thereby enhancing chemotherapy sensitivity (Nishitani et al., 2013).

BCAA metabolism plays a crucial role in cancer resistance to chemotherapy, targeted therapy, and immunotherapy, as illustrated in the figure. By upregulating BCAAs transaminases (such as BCAT1 and BCAT2) (Günther et al., 2022; Hutson et al., 1998; Ma et al., 2022),cancer cells enhance their metabolic activity, promoting growth and reducing sensitivity to chemotherapy drugs. BCKDH, as the rate-limiting step in BCAA metabolism, has been the focus of ongoing development for inhibitors targeting its activity (East et al., 2021; Roth Flach et al., 2023). Leucine, as an activator of the mTOR pathway, strengthens mTOR signaling, contributing to resistance against targeted therapies. Additionally, some studies have reported that miRNAs can target BCATs to exert antitumor effects (Ma et al., 2022). Furthermore, BCAA metabolism depletes resources needed by T cells, suppressing immune responses and leading to immunotherapy resistance. Inhibiting BCAA metabolism (such as using BCAT1 inhibitors) can reverse resistance and enhance cancer cells’ sensitivity to chemotherapy, targeted therapy, and immunotherapy (Table 2). Combining BCAA metabolism inhibitors with existing therapies may be an effective strategy to overcome resistance and improve the efficacy of cancer treatments.

**TABLE 2:** BCAAs resistance-related targets.

Cancer type	Inhibitor	Mechanism of drug resistance	Drug resistance target
NSCLC (Non-small cell lung cancer)	BCAT1 inhibitor WQQ-345 (Zhang et al., 2024a)	Glycolysis	Osimertinib
Liver cancer	BCAT1 inhibitor (Nishitani et al., 2013)	Inhibition of mTOR complex 2	5-FU
Glioblastoma	BCAT1 inhibitor (Bagchi et al., 2021)	BCKDK-BCAT1	Temozolomide (TMZ)
Breast cancer	LAT1 inhibitor JPH203 (Stine et al., 2022)	Leucine uptake and mTORC1 signaling	Aromatase inhibitors (AI)
Breast cancer	BCKDK inhibitor (Ibrahim et al., 2023)	mTORC1-Aurora axis	Paclitaxel
Breast cancer	PELP1 (Proline, Glutamic acid, Leucine-rich Protein 1) (Gonugunta et al., 2014)	Serine/Threonine protein kinase mTOR	Estrogen receptor (ER)
Bladder cancer	LRFN2 (Yu et al., 2023)	Anti-tumor immunity	PD-1 immunotherapy
Breast cancer	BCKDK inhibitor (Biswas et al., 2021)	Inhibition of protein translation, mitochondrial dysfunction	Doxorubicin
AML (Acute Myeloid Leukemia)	BCAT1 (Tosello et al., 2024)	Regulation of histone methylation	PARP inhibitor
Refractory T-ALL	BCAT1 inhibitor (Wang et al., 2024b)	Key transcription factor regulating autophagy	Elthrombopag targeting TFEB

## 4 Summary

BCAAs play a crucial role in cellular metabolism, signaling, and energy supply. In cancer development and progression, BCAA metabolism exerts a complex regulatory function, and due to the distinct genetic backgrounds of different tumors, BCAAs metabolic reprogramming manifests in varying patterns across different cancers. This presents a novel therapeutic approach for cancer treatment in the future. For instance, non-small cell lung cancer shows increased BCAAs uptake, while pancreatic cancer exhibits decreased BCAAs consumption, and the metabolic enzymes involved also behave differently. The dependency on the two BCAT isoenzymes, BCAT1 and BCAT2, differs across cancers. Targeting the specific metabolic reprogramming patterns of BCAAs in tumors could enable the design of precision therapies.

In the tumor immune microenvironment, immune cells and tumor cells compete for BCAAs uptake, which weakens the cytotoxic activity of immune cells and diminishes the immune microenvironment’s effectiveness. Enhancing the antitumor activity of immune cells by increasing relevant BCAA metabolism without promoting tumor-associated metabolic reprogramming is a key challenge. Novel treatments like chimeric antigen receptor (CAR)-T cell therapy offer a promising approach by altering metabolic targets in T cells to improve immune cell cytotoxicity in the tumor microenvironment.

While cancer treatment strategies have become increasingly common, resistance to therapies often reduces the efficacy of these treatments. Our study explored how cancer-related resistance could be reversed by targeting BCAA metabolism, enhancing sensitivity to radiotherapy, chemotherapy, targeted therapy, and immunotherapy. Excitingly, some studies have shown that immune checkpoint inhibitors, such as PD-1/PD-L1 inhibitors, can boost T cell activity and overcome BCAA-induced immune suppression. Combining these inhibitors with BCAAs metabolic inhibitors has demonstrated stronger antitumor effects.

In advanced stages of cancer, patients often experience severe nutrient depletion, leading to symptoms like cachexia, where muscle metabolism also depends on certain BCAA-related enzymes. However, the potential side effects of BCAA metabolic enzyme inhibitors on normal tissues remain a concern. Thus, achieving tumor-specific precision targeting of BCAA metabolism is crucial. Looking ahead, the development of more precise treatments targeting BCAA metabolism holds promise for offering new directions and therapeutic models in cancer treatment.
